# Simultaneous Detection of Missing Amphibians and Their Fungal Pathogen in a Biodiversity Hotspot Using eDNA

**DOI:** 10.1111/mec.70075

**Published:** 2025-08-08

**Authors:** Carla Martins Lopes, C. Guilherme Becker, Délio Baêta, Juliane Petri de Carli Monteiro, Mariana Lúcio Lyra, Kelly Raquel Zamudio, Anthony Chariton, Célio Fernando Baptista Haddad

**Affiliations:** ^1^ Departamento de Biodiversidade and Centro de Pesquisa em Biodiversidade e Mudanças do Clima Universidade Estadual Paulista (UNESP) Rio Claro São Paulo Brazil; ^2^ Department of Biology The Huck Institutes of the Life Sciences, The Pennsylvania State University University Park Pennsylvania USA; ^3^ Center for Infectious Disease Dynamics, One Health Microbiome Center The Huck Institutes of the Life Sciences, The Pennsylvania State University University Park Pennsylvania USA; ^4^ New York University Abu Dhabi Abu Dhabi UAE; ^5^ Department of Integrative Biology The University of Texas at Austin Austin Texas USA; ^6^ Enviromental (e)DNA and Biomonitoring Lab, School of Natural Sciences Macquarie University Sydney New South Wales Australia

**Keywords:** *Batrachochytrium dendrobatidis*, biomonitoring, Brazilian Atlantic forest, environmental DNA, metabarcoding, threat

## Abstract

Since the 1970s, striking amphibian declines in population abundances and presumed extinctions have been recorded globally. The chytrid fungus *Batrachochytrium dendrobatidis* (*Bd*) is one of the key drivers of these declines. To investigate the potential role of *Bd* in the decline of Brazil's amphibians, we first used eDNA metabarcoding to survey for DNA traces of 10 threatened amphibian species from the Southern Brazilian Atlantic forest (SBAF). Using the eDNA samples of this survey, along with eDNA samples from previous surveys of 32 threatened amphibian species from the Northern Brazilian Atlantic forest (NBAF), we examined the relationships between the presence or absence of targeted amphibians with the presence or absence of *Bd* lineages. We detected DNA traces of two of the 10 targeted species in the survey of SBAF, including one species that was missing for over 40 years ( *Cycloramphus cedrensis* ). We also detected DNA traces of two *Bd* lineages (*Bd*‐GPL and *Bd*‐Brazil) at nine of the 12 sampling sites where community‐level amphibian declines were first reported in the late 1970s. Our results support a post‐panzootic scenario, where some threatened amphibian species are coping to persist in the presence of enzootic *Bd*. Our research also provides novel insights into simultaneous eDNA surveys targeting both hosts and pathogens, offering important methodological contributions to tropical ecology and conservation.

## 1 Introduction

The Anthropocene is resulting in accelerated losses of global biodiversity (Johnson et al. 2017). Among all Chordata evaluated by the red list of the International Union for Conservation of Nature (Re:wild, Synchronicity Earth, IUCN SSC Amphibian Specialist Group 2023), amphibians have one of the highest proportions of formally threatened species (41%); a pattern largely explained by their high sensitivity to environmental degradation and emerging diseases (Wake and Vredenburg 2008). With 1188 described species, Brazil has the highest amphibian diversity of any country, with most of these (60.52%) occurring in the Brazilian Atlantic forest (Figueiredo et al. 2021). From 1976 to 1985, the Brazilian Atlantic forest experienced a peak in the amphibian decline crisis (Toledo et al. 2023). Recent research supports a range of factors likely driving this ‘amphibian crisis’, including the synergistic effects of climate change, habitat degradation, and the fungal pathogen *Batrachochytrium dendrobatidis* (*Bd*), mainly represented by the Global Panzootic Lineage (*Bd*‐GPL), which has been associated with disease‐induced declines globally (Becker et al. 2023; Carvalho et al. 2017; Eterovick et al. 2005). Most of the affected species in Brazil have not shown signs of recovery (Toledo et al. 2023), and a number of species only known from their type series (or specimen) have never been observed again (Lopes et al. 2021, 2023). Consequently, there are significant knowledge gaps about the geographic distributions, population dynamics, and phylogenetic relationships for many Brazilian amphibian species that suffered the impact of this crisis. Addressing these shortfalls is critical for assessing the threats to amphibians, thereby guiding targeted conservation efforts.

Ecological surveys are crucial for monitoring the population status and persistence of species. However, surveying elusive species with low population abundances in remote tropical locations is challenging, making biomonitoring costly and time‐consuming (Zinger et al. 2020). The detection of DNA traces left by organisms in the environment (eDNA) is a highly sensitive, relatively rapid, non‐invasive (no handling or tissue sampling), and non‐destructive approach which is being increasingly used to survey and monitor a wide range of taxa and communities across many ecosystems (Taberlet et al. 2018). eDNA has the capacity to overcome some of the challenges associated with traditional field survey methods, allowing the detection of organisms that are difficult to sample or morphologically identify in the field (Lopes et al. 2017; Taberlet et al. 2018). In Brazil, recent studies have demonstrated the potential of eDNA metabarcoding to comprehensively survey amphibian communities, providing critical ecological data to aid in the protection of the Neotropical herpetofauna (Lopes et al. 2017, 2021, 2023). However, this approach has not been widely used to survey hosts and pathogens simultaneously, either during or after epizootic outbreaks.

The objective of this study was to examine the relationships between the occurrence or absence of targeted threatened amphibians and the presence of *Bd* lineages. First, new eDNA samples were obtained by surveying five sites within the Southern Brazilian Atlantic forest, targeting 10 amphibian species of conservation concern. Second, we used the amphibian presence/absence data and the eDNA samples obtained from this study and two previous studies targeting 32 species of amphibians from seven localities in the Northern Brazilian Atlantic forest, to concurrently search for the presence of any of the five major *Bd* lineages known to date (O'Hanlon et al. 2018). Specifically, our aims were to: (i) determine whether targeted amphibian species with historical records of declines or disappearances are still present in the Brazilian Southern Atlantic forest; (ii) improve our knowledge about the extant geographic distribution and habitat use of these amphibians; and (iii) correlate spatial occurrence of any detected *Bd* lineage with DNA traces of the 42 targeted amphibians from both the Southern and Northern Brazilian Atlantic forests. By examining whether there is a positive or negative association between *Bd* and DNA traces from our target amphibian species in the environment, we hope to infer whether some populations may be adapting to persist alongside the pathogen – potentially through resistance or tolerance mechanisms. Collectively, we anticipate that this information will expand our understanding of the potential drivers of host‐pathogen co‐occurrence and the resilience of threatened amphibian populations.

## 2 Materials and Methods

### 2.1 Target Amphibian Species

**FIGURE 1:**
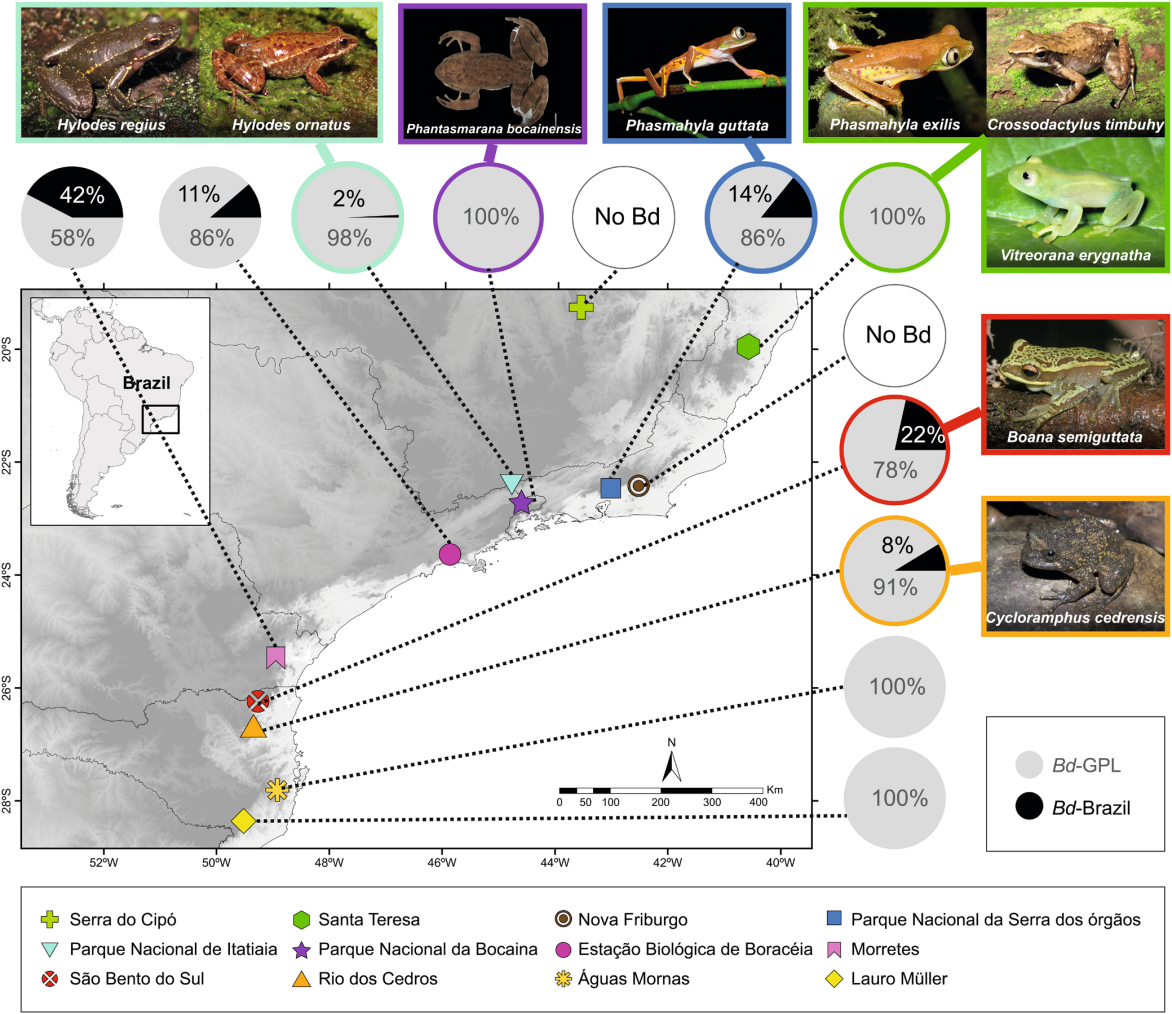
Sampling sites surveyed for the presence of 42 threatened amphibian species and *Batrachochytrium dendrobatidis* lineages in the Southern (Morretes, São Bento do Sul, Rio dos Cedros, Águas Mornas, and Lauro Müller) and Northern (Serra do Cipó, Santa Teresa, Nova Friburgo, Parque Nacional da Serra dos Órgãos, Parque Nacional de Itatiaia, Parque Nacional da Bocaina, and Estação Biológica de Boracéia) Brazilian Atlantic forest and adjacent Cerrado grasslands. Amphibians positively detected in this ( *Boana semiguttata* and *Cycloramphus cedrensis* ) and our previously published studies ( *Hylodes ornatus* , *Hylodes regius* , *Crossodactylus timbuhy* , *Vitreorana eurygnatha* , *Phasmahyla exilis* , *Phasmahyla guttata* , and *Phantasmarana bocainensis*), and the proportion of sequence reads of *Bd* lineages (*Bd*‐GPL and *Bd*‐Brazil) recovered at each sampling site are shown. Photo credits: CFB Haddad ( *C. timbuhy* , *H. regius*, and *V. eurygnatha* ), D Baêta ( *C. cedrensis* ), FP de Sá ( *H. ornatus* ), LR Malagoli ( *P. guttata* ), JL Gasparini ( *P. exilis* ) MNRJ (*P. bocainensis*), and T Condez (*Boana semiguttata*).

In the present study, we assembled a list of 10 amphibian species distributed across five localities in southern Brazil (hereafter referred to as the southern Brazilian Atlantic forest – SBAF) (Table 1 and Figure 1), by consulting reports of declines in the literature, amphibian collections in Brazil, and the Species Link database (http://www.splink.org.br/). All 10 targeted species are aquatic‐breeders which have suffered marked declines in population abundances compared to historical baseline records; or the species have not been recorded by any survey method at their type localities (locally disappeared) or throughout their geographical distribution (globally disappeared) for more than 10 years (Table 1).

**TABLE 1:** List of 10 amphibian species of conservation concern in the Southern Brazilian Atlantic forest (SBAF) targeted in this study and 32 amphibian species from the Northern Brazilian Atlantic forest (NBAF) targeted in previous studies (Lopes et al. 2021 and 2023). For each species we list the last year the species was recorded at the sites surveyed, how species were classified in the study, the conservation status according to the IUCN red list, and if the DNA of species was detected (Y) or not (N) in the samples.

Sites	Family	Species searched	Last observ.	Classif.	IUCN	eDNA detection
Morretes – PR (SBAF)	Cycloramphidae	*Cycloramphus duseni*	1911^a^	GD	DD	N
		*Cycloramphus mirandaribeiroi*	2013^b,c^	GD	DD	N
	Hylidae	*Bokermannohyla langei*	1978^d^	GD	DD	N
São Bento do Sul – SC (SBAF)	Cycloramphidae	*Cycloramphus diringshofeni*	1952^e^	GD	DD	N
	Hylidae	*Boana semiguttata*	2013^f^	LD	LC	Y
		*Phrynomedusa appendiculata*	1924^g^	LD	NT	N
Rio dos Cedros – SC (SBAF)	Cycloramphidae	*Cycloramphus cedrensis* *	1982^h,d^	GD	DD	Y
Águas Mornas – SC (SBAF)	Cycloramphidae	*Cycloramphus asper*	2014^b^	GD	DD	N
		*Cycloramphus catarinensis*	1905^d^	LD	DD	N
Lauro Müller – SC (SBAF)	Cycloramphidae	*Thoropa saxatilis*	1979^i^	GD	NT	N
	Hylidae	*Phrynomedusa appendiculata*	1970^h^	LD	NT	N
Parque Nacional da Serra do Cipó, MG (NBAF)	Hylidae	*Scinax pinimus* *	2016^j^	PD	DD	N
Santa Teresa, ES (NBAF)	Aromobatidae	*Allobates capixaba*	1983^b^	LD	NA	N
	Centrolenidae	*Vitreorana eurygnatha*	2017^k^	PD	LC	Y
	Cycloramphidae	*Cycloramphus fuliginosus*	1981^l^	LD	LC	N
	Hylidae	*Phasmahyla exilis*	2019^k^	PD	LC	Y
		*Phrynomedusa marginata*	1988^b^	GD	LC	N
	Hylodidade	*Crossodactylus gaudichaudii*	1981^l^	LD	LC	N
		*Crossodactylus timbuhy* *	2018^k^	PD	NA	Y
		*Hylodes babax*	1981^l^	LD	DD	N
Nova Friburgo, RJ (NBAF)	Leptodactylidae	*Crossodactylodes pintoi*	1909^m^	GD	DD	N
Parque Nacional da Serra dos Órgãos, RJ (NBAF)	Bufonidae	*Dendrophryniscus organensis*	2012^n^	PD	NA	N
	Cycloramphidae	*Cycloramphus ohausi*	1977^d^	GD	DD	N
		*Thoropa petropolitana*	1982^b^	GD	VU	N
	Hylidae	*Aplastodiscus musicus*	2016^o^	PD	DD	N
		*Boana claresignata*	1964^p^	GD	DD	N
		*Phasmahyla guttata*	2016^q^	PD	LC	Y
		*Phrynomedusa vanzolinii*	1929^r^	GD	DD	N
Parque Nacional de Itatiaia – RJ (NBAF)	Hylidae	*Scinax obtriangulatus*	2009^j^	LD	LC	N
	Hylodidade	*Crossodactylus grandis*	1977^d^	GD	DD	N
		*Crossodactylus werneri*	1978^s^	GD	NA	N
		*Hylodes glaber*	1978^c^	GD	DD	N
		*Hylodes ornatus* *	2012^t^	PD	LC	Y
		*Hylodes regius* *	2016^j^	PD	DD	Y
	Leptodactylidae	*Paratelmatobius lutzii*	1978^u^	GD	DD	N
	Strabomantidae	*Holoaden bradei*	1978^c^	GD	CR	N
Parque Nacional da Serra da Bocaina – SP (NBAF)	Hylidae	*Boana clepsydra*	1980^p^	GD	DD	N
	Hylodidade	*Phantasmarana bocainensis*	1968^v^	GD	DD	Y
Estação Biológica de Boracéia – SP (NBAF)	Centrolenidae	*Vitreorana eurygnatha*	1979^x^	LD	LC	N
	Cycloramphidae	*Cycloramphus boraceiensis*	1979^x^	LD	LC	N
		*Cycloramphus semipalmatus*	1979^x^	LD	NT	N
		*Thoropa taophora*	1979^x^	LD	NA	N
	Hylidae	*Phrynomedusa vanzolinii*	1973^r^	GD	DD	N
	Hylodidade	*Crossodactylus dispar*	1977^s^	LD	DD	N
		*Hylodes asper*	1979^x^	LD	LC	N

*Note:* Brazilian States: PR – Paraná; SC – Santa Catarina; ES – Espírito Santo; MG – Minas Gerais; RJ – Rio de Janeiro; SP – São Paulo. Species classification in this study: PD – population declined; LD – species locally disappeared ≥ 10 years; and GD – species globally disappeared ≥ 10 years. IUCN codes: CR – Critically Endangered; VU – Vulnerable; NT – Near Threatened; LC – Least Concern; DD – Data Deficient; and NA – Not Assessed. Reference for the information: ^a^Andersson (1914); ^b^Museu Nacional (MNRJ), Universidade Federal do Rio de Janeiro; ^c^Museu de Diversidade Biológica (ZUEC‐AMP), Universidade Estadual de Campinas; ^d^Smithsonian's National Museum of Natural History (NMNH); ^e^Lingnau et al. (2008); ^f^Universidade Federal de Minas Gerais (UFMG); ^g^Baêta et al. (2016); ^h^Museu de Zoologia (MZUSP), Universidade de São Paulo; and ^i^Crocroft and Heyer (1988); ^j^Coleção Célio Fernando Baptista Haddad (CFBH), Universidade Estadual Paulista; ^k^Museu de Biologia Professor Mello Leitão (MBML), Instituto Nacional da Mata Atlântica; ^l^Weygoldt (1989); ^m^Santos et al. (2020); ^n^Coleção Herpetológica do Departamento de Zoologia, Universidade Federal do Rio de Janeiro (ZUFRJ); ^o^Bezerra et al. (2020); ^p^Lyra et al. (2020); ^q^Laboratório de Biossistemática de Anfíbios (UNIRIO), Universidade Federal do Estado do Rio de Janeiro; ^r^Cruz (1991); ^s^Pimenta et al. (2014); ^t^de Sá et al. (2015); ^u^Pombal and Haddad (1999); ^v^Giaretta et al. (1993); ^x^Heyer et al. (1990).

* Target species audio‐visually observed in the field during eDNA sampling.

To examine the correlations between the occurrence/absence of targeted amphibians and *Bd* lineages, additional data and eDNA extracts from a previous survey sampling seven localities from the Northern Brazilian Atlantic forest (NBAF) were also used (Lopes et al. 2021, 2023). This previous study targeted 32 amphibians of high conservation concern, with only seven species being detected using eDNA (Table 1).

The perimeter and polygon area of each site surveyed and the linear distance between the southernmost and northernmost samples collected were estimated using Google Earth Pro 7.3.6.9796.

### 2.2 Environmental DNA Sampling and Laboratory Procedures

The new eDNA samples from the SBAF (*N* = 48) were collected between March and April 2019, while the samples from NBAF (*N* = 69) were obtained between December 2015 and February 2017 (Appendix S1). For both surveys, sampling was timed with the end of the breeding season for most of the targeted species (September–March), coinciding with warmer days and longer photoperiods. In total, 117 water samples were obtained from a diverse array of environments, including: streams; rivers; puddles; ponds; swamps; rocky seeps; and bromeliads (Appendix S1). The sites captured the potential areas of distribution and exact known spots of historical occurrence for our targeted species (Table 1 and Appendix S1). Water filtering was carried out in the field following the protocols of Lopes et al. (2017, 2021, 2023), using Envirochek HV 1 μm (Pall Corporation, Port Washington, USA), VigiDNA 0.45 μm (SPYGEN, Le Bourget‐du‐Lac, France), or Sterivex 0.22 μm (Millipore, Burlington, USA) sampling capsules, depending on the source of water sampled (Appendix S1). The volumes of sampled water (30 L [median], 1–120 L [2.5%–97.5% percentiles, respectively], Appendix S1) varied with the source of water and filters used. Smaller water volumes were obtained from rocky seeps, bromeliads, puddles, ponds, or water sources with high loads of suspended material, with the latter hindering the filtering of large volumes due to the potential for filter clogging. After filtering, capsules were filled with lysis buffer (Tris–HCl 0.1 M, EDTA 0.1 M, NaCl 0.01 M and N‐lauroyl sarcosine 1%, pH 7.5–8) and stored at room temperature until DNA extraction. In cases where only small volumes of water (15 mL) were obtained, the filtration step was excluded, with the DNA extracted directly from water samples (Lopes et al. 2023).

Total DNA was extracted using the DNeasy Blood & Tissue Extraction Kit (Qiagen GmbH, Hilden, Germany) (Lopes et al. 2017, 2021, 2023). For the detection of threatened amphibian species, ~50 bp of the 12S rRNA mitochondrial gene were amplified using the primers batra_F (5′‐ACACCGCCCGTCACCCT‐3′) and batra_R (5′‐GTAYACTTACCATGTTACGACTT‐3′) (Valentini et al. 2016). Reactions were prepared for a final volume of 15 μL, using 1.5 μL of DNA extract, 1X AmpliTaq Gold 360 Master Mix (Life Technologies, Carlsbad, CA, USA), 10% Enhancer MM (Life Technologies, Carlsbad, CA, USA), and 0.2 μM of each primer. The PCR conditions were 95°C for 10 min, followed by 55 cycles of 95°C for 30 s, 55°C for 30 s, 72°C for 1 min, and a final step of 72°C for 7 min. PCR negative controls (containing DNA‐free water) and positive controls were used to monitor possible contamination and to estimate the detection power of our approach. Two positive control communities (POS_01 and POS_02) were assembled. POS_01 consisted of Brazilian amphibian species ( *Hylodes cardosoi* , *Cycloramphus cf cedrensis*, *Scinax catharinae* , *Hylodes perplicatus* , and *Boana poaju* ) that might occur in the sampling sites surveyed and could be detected in the eDNA samples. POS_02 consisted of Australian species ( *Crinia signifera* , *Litoria verreauxii* , and *Limnodynastes dumerilii* ) which do not occur in Brazil. Collectively, positive control communities enabled the tracking of potential contaminations between eDNA samples during PCR pipetting. Twelve PCR amplifications were carried out for all eDNA samples and controls.

The DNA extracts for the 117 samples were also examined for the presence of any *Bd* lineage. To do this, a ~90 bp region encompassing the 5.8S rRNA gene and a portion of the flanking internal transcribed spacer 1 (ITS1) were amplified using the primers *Bd*1a (5′‐CAGTGTGCCATATGTCACG‐3′) (Annis et al. 2004) and 5.8S Chytr (5′AGCCAAGAGATCCGTTGTCAAA3′) (Boyle et al. 2004). Reactions were prepared for a final volume of 15 μL, using 1.5 μL of DNA extract, 1X AmpliTaq Gold 360 Master Mix (Life Technologies, Carlsbad, CA, USA), 10% Enhancer MM (Life Technologies, Carlsbad, CA, USA), and 0.5 μM of each primer. The PCR conditions were 95°C for 5 min, followed by 50 cycles of 95°C for 15 s, 60°C for 30 s, 72°C for 1 min, and a final step of 72°C for 7 min. Extraction and PCR negative controls (containing DNA‐free water) and positive controls (containing DNA of *Bd*‐GPL [Flinders#5] and *Bd*‐Brazil [CLFT068] lineages) were amplified for monitoring possible contamination and to estimate the detection power of our approach. Four PCR amplifications were carried out for all eDNA samples and controls.

All forward and reverse primers used for amphibians and *Bd* amplifications were 5′ labelled with a unique eight‐bp molecular index for each PCR replicate, to allow the assignment of sequence reads to appropriate PCR replicates during the sequence filtering process. All PCR products were purified using the MinElute PCR purification kit (Qiagen GmbH, Hilden, Germany) and quantified on a Qubit (Qiagen GmbH, Hilden, Germany). For the previously published NBAF amphibian amplifications, paired‐end sequencing (2 × 125 bp) was carried out using Illumina HiSeq 2500 (Illumina, San Diego, CA, USA), at Fasteris (Geneva, Switzerland). For the new SBAF amphibian amplifications, paired‐end sequencing (2 × 75 bp) was carried out using Illumina MiSeq v3 sequencer (Illumina, San Diego, CA, USA), at Ramaciotti Centre for Genomics (Sydney, Australia). The paired‐end sequencing (2 × 150 bp) of *Bd* amplifications was carried out using MiniSeq (Illumina, San Diego, CA, USA), at EcoMol Consultoria (Piracicaba, Brazil), following the manufacturer's instructions.

### 2.3 Reference Databases

A custom sequence reference database was assembled to improve the taxonomic assignment of the amphibian sequences recovered from the eDNA samples. The protocols and the list of 161 species sequenced for the reference database of NBAF localities are described in Lopes et al. (2021). For the SBAF, we compiled 153 tissue samples of 71 amphibian species known to occur now or in the past at each surveyed site (data available at https://datadryad.org/stash/share/ueUEipLVBGB45Y9abHERoXLWdjPeYnjEdfUzJhINObE). Tissues for the reference database were sourced from the Célio F. B. Haddad tissue collection (CFBHT) at Universidade Estadual Paulista, Rio Claro, São Paulo, Brazil; Museu Nacional – Universidade Federal do Rio de Janeiro (MNRJ), Rio de Janeiro, Rio de Janeiro, Brazil; Museu de Zoologia (MZUSP) at Universidade de São Paulo, São Paulo, São Paulo, Brazil; and Museu de Diversidade Biológica – Universidade Estadual de Campinas (ZUEC‐AMP), Campinas, São Paulo, Brazil. For target species for which no tissue samples were available, sequences of closely related congeners were included in the database.

Total DNA from 10 mg of tissue samples of amphibians was extracted using a standard high‐salt protocol (Lyra et al. 2017). The 12S rRNA mitochondrial gene was amplified using the primers MVZ59 (5′ATAGCACGTAAAAYGCTDAGATG3′) (Graybeal 1997) and tVal (5′TGTAAGCGARAGGCTTTKGTTAAGCT3′) (Wiens et al. 2005), following the protocols described in Lopes et al. (2017). PCR products were purified using Exonuclease I and Shrimp Alkaline Phosphatase, following the guidelines of the suppliers. Both DNA strands were sequenced. Consensus sequences were visually inspected, edited, and constructed using Geneious 7.1.3 (Kearse et al. 2012). The final 12S metabarcoding reference database was assembled using the relevant part of the 12S rRNA mitochondrial sequences of amphibians extracted from the EMBL database (*vrt* release 143) and the custom reference database assembled by us.

To identify the *Bd* lineages, a sequence reference database was constructed by downloading from the EMBL (downloaded on 21/10/2022) the relevant part of the 5.8S rRNA and ITS1 sequences of all *Bd* lineages already genotyped and properly identified in previous studies (Byrne et al. 2019; Miller et al. 2018; O'Hanlon et al. 2018; Schloegel et al. 2012). The *Bd* lineages present in the sites surveyed were determined by comparing each of the sequences recovered from our eDNA samples to the sequences of *Bd* lineages in the reference database.

Both amphibian and *Bd* metabarcoding reference databases were assembled using programs in ecoPCR 0.5.0 (Ficetola et al. 2010) and OBITools 1.2.13 (Boyer et al. 2016) packages.

### 2.4 eDNA Sequence Filtering and Taxonomic Assignment

Recovered sequences were filtered and taxonomically annotated using ecoPCR 0.5.0, OBITools 1.2.13, and R 4.2.3 (R Development Core Team 2022), following the procedures described in Lopes et al. (2021). Paired‐end reads were assembled to construct consensus sequences and assigned to their appropriate PCR replicates, allowing 2 bp mismatches for each primer and no error for molecular indexes. All sequences with total read counts ≤ 10 among all PCR replicates and which were shorter than 20 bp and 50 bp for amphibians and *Bd* datasets, respectively, were removed. The taxonomic identity for each sequence was assigned using the reference databases assembled for each specific molecular marker. When two or more species were assigned to a given eDNA sequence, only the species that is known to occur in the region studied, following the current taxonomy (Frost 2023), was considered. A directed acyclic graph (DAG) was constructed to identify sequences in each PCR replicate as “head” (the most frequent sequence within a group of sequences differing by a single indel/substitution), “internal” (less frequent sequences within the group of related sequences), or “singleton” (sequence not related to other sequence variants). Sequences identified as “internal” were removed from downstream analyses, as these are most likely PCR artefacts (Taberlet et al. 2018).

Profiles of eDNA samples were compared to the negative and positive controls as means of eliminating potential contaminants, chimeras, and PCR/sequencing errors from each dataset. Sequences with a frequency of occurrence ≤ 0.001 or 0.1264 per PCR replicate depending on the NBAF dataset, and ≤ 0.01 and ≤ 0.02 per PCR replicate for SBAF and *Bd* samples, respectively, were removed from the datasets. The thresholds were determined based on the profile of positive controls to ensure that, after filtering, all relevant detectable sequences would be retained while most low‐frequency noisy reads would be eliminated. eDNA sequences with identity level with sequences from the reference database ≤ 96% for amphibians and ≤ 98% for *Bd* sequences; sequences with maximal average read counts in negative controls; and dysfunctional PCRs with low sequencing depth, as ≤ 200 or 920 reads depending on the NBAF dataset, and ≤ 400 and ≤ 3300 reads for SBAF and *Bd* samples, respectively, were also removed. For the *Bd* dataset, potential tag jump bias was also dealt with and eliminated from further analysis by excluding from each PCR replicate sequences with frequency ≤ 0.05 compared to all PCR replicates; and sequences that appeared in only one out of four PCR replicates of each sample.

Bioinformatic pipelines for filtering and taxonomic annotation of sequences are deposited in Dryad (https://datadryad.org/stash/share/ueUEipLVBGB45Y9abHERoXLWdjPeYnjEdfUzJhINObE).

### 2.5 Statistical Analyses

Specificity, amplification success, and mismatch rates were evaluated to verify whether the primers effectively amplify target amphibian species and *Bd* lineages in Brazil. The detection, or lack thereof, of sequences assigned to more than one species was carefully double‐checked to prevent incorrect assumptions based on false detections or unnoticed detection of target species resulting from occasional limitations in the marker's taxonomic resolution. The proportion of sequence reads for each amphibian taxon or *Bd* lineage was calculated based on the sum of read counts among PCR replicates of each eDNA sample. Comparisons between the prevalence of *Bd* lineages recovered were performed by examining the number of sequence reads and samples positively detected at each locality, using the Wilcoxon signed‐rank test, under a significance of *p* ≤ 0.01.

False positive rates (p10), the probability of detection (p11), and site occupancy (psi) were estimated for each target amphibian species and *Bd* lineage to evaluate the confidence on taxa presences/absences in the sites surveyed. Matrices of presence/absence were then constructed considering the PCR replicates of each eDNA sample, within each locality surveyed for the target amphibian species detected with eDNA, or the overall data for *Bd* lineages. The JAGS program (Plummer 2003) in the package R2jags (Su and Yajima 2024) was used to apply the site occupancy–detection Bayesian inference model of Royle and Link (2006), considering the prior of maximum probability of false presences as 0.01 and 0.02 (based on positive control results), for amphibians and *Bd* data, respectively, running four chains of 100 000 iterations, 50 000 as burn‐in, and saving 1000 iterations per chain. For more details, see Lahoz‐Monfort et al. (2015).

We used pairwise Pearson correlations to assess the strength of associations among all focal biotic and abiotic variables. We followed this initial screening step with Generalised Linear Mixed Models (GLMMs) with Logistic Link to explore which variables best explained the weighted proportion of target amphibian species detected in water samples through eDNA. We included as explanatory variables filtered water volume, presence of each *Bd* lineage detected, and the following environmental variables extracted at 1 km resolution from Bioclim2 (Fick and Hijmans 2017): elevation, annual temperature (BIO1), and annual precipitation (BIO12). All possible models were generated, and the most parsimonious, i.e., the one with the lowest AIC score, explaining our single response variable (proportion of target amphibian species detected in water samples) was identified. Two additional variables were also included as random effects, which were not subject to pruning, in our model selection: sampling locality and target amphibian species. The number of samples (capsules) per sampling site was weighted in all analyses. A similar model selection approach was used, which included each *Bd* lineage detected as the single response variable in separate model selection pipelines, while detection of target amphibian species was treated as an explanatory variable along with the same environmental variables described above. These analyses were performed with a binomial distribution and logit link using the R package GLMMTMB, simultaneously testing for cross‐correlation among explanatory variables using Variance Inflationary Factor (VIF), and under a significance threshold of *p* ≤ 0.05.

## 3 Results

The batra primers showed no mismatches with the 12S rRNA reference sequences for the target amphibians and were able to distinguish 61.24% of all amphibian species represented in the final 12S reference database. Most of the target species we were seeking to detect showed unique (and therefore diagnostic) 12S fragments. We had three target species that did not have unique sequences and shared 100% identity with another non‐target species. *Cycloramphus cedrensis* and *Cycloramphus izecksohni* shared 100% sequence identity, as did *Cycloramphus fuliginosus* / *Cycloramphus faustoi* and *Scinax obtriangulatus* / *Scinax hiemalis* . This lack of diagnostic power is the result of closely related species and, in some cases, poorly defined taxonomies. However, in our case, this did not affect our inferences because we did not detect DNA of *Cycloramphus fuliginosus* or *Scinax obtriangulatus* in any of our samples. The species we did detect ( *Cycloramphus cedrensis* ) is part of a species complex with *C. izecksohni* with poor taxonomic boundaries, so we can infer that the animal present was one of those two species. All other target anurans were identified by our match criteria at the 96% level match or higher at the barcoding fragment. The primers used to amplify *Bd* sequences had only one mismatch with a single *Bd* sequence in our final reference database, identified as the ZFT‐CU1 *Bd* strain, which does not occur in Brazil.

We obtained a total of 11 185 085 consensus sequence reads across the eDNA samples and controls for SBAF amphibian dataset (Appendix S2). After the sequence filtering process, only the relevant sequences were retained in positive controls, and no sequences remained in extraction and PCR negative controls for amphibian datasets.

Overall, 48 (Molecular Operational Taxonomic Units) MOTUs associated with amphibians were identified among all SBAF sampling sites, from which we identified 17 species from 14 genera and seven families. Eight MOTUs were identified to the level of the tribe Cophomantini (Appendix S3). At Morretes, we detected 19 MOTUs, assigned to 13 taxa, none of which corresponded to our target species (Appendix S3). At São Bento do Sul, we detected 20 MOTUs, assigned to 13 taxa: one of these MOTUs had a 100% identity match with a sequence corresponding to the target species *Boana semiguttata* (Figure 1 and Appendix S3). At Rio dos Cedros, we detected eight MOTUs, assigned to seven taxa; one of these MOTUs had a 100% identity match with sequences of both *Cycloramphus cedrensis* (a target species) and *C. izecksohni* (not a target species, not known to occur at Rio dos Cedros, and part of a species complex with *C. cedrensis* ; Figure 1 and Appendix S3). At Águas Mornas, we detected eight MOTUs, assigned to seven taxa, none of which corresponded to our target species. At Lauro Müller, we detected 13 MOTUs, assigned to 10 taxa, none of which corresponded to our target species. Hence, only two of the target 10 species were detected, *Boana semiguttata* and *Cycloramphus cedrensis* . Details on all detected taxa, both targeted and non‐targeted species, are provided in Appendix S3.

*Boana semiguttata* (Figure 1) is known to be distributed from Palmeira and Piraquara, in the State of Paraná, to São Francisco de Paula, in the State of Rio Grande do Sul (Faivovich et al. 2021). This species is still present in large parts of its distribution, but it was last registered at the type locality in Rio Vermelho, São Bento do Sul, in the State of Santa Catarina 10 years ago. We found DNA traces of *B. semiguttata* in two eDNA samples near São Bento do Sul (SBS_07 and SBS_08), one sample from São Bento do Sul close to Rio Vermelho (SBS_01), and one sample from Rio Vermelho (SBS_02) (Appendix S1). However, we did not observe any individuals corresponding to *B. semiguttata* during our fieldwork.

*Cycloramphus cedrensis* (Figure 1) is only known from the type locality, indicated in the original description as “Santa Catarina, 12 km E of Rio dos Cedros on the road to Rio São Bernardo, approximately 26°44′ S, 49°20′ W” (Heyer 1983). The species was last seen 40 years ago. During our field sampling, at the site corresponding to the eDNA sample CED_02 (Appendix S1), we collected tadpoles, juveniles, adult females, and males of a *Cycloramphus* species that corresponds to the description of *C. cedrensis* . Morphological and further DNA sequence data are currently being collected to determine whether *C. cedrensis* and *C. izecksohni* are synonymous species, to inform further conservation actions for the remaining populations.

In the previous surveys in NBAF (Lopes et al. 2021, 2023) DNA traces of seven of the 32 target amphibian species were detected: four species with signals of declines in population abundance from Parque Nacional de Itatiaia ( *Hylodes ornatus* and *Hylodes regius* ) and Santa Teresa ( *Crossodactylus timbuhy* and *Vitreorana eurygnatha* ); two species locally disappeared from Santa Teresa ( *Phasmahyla exilis* ) and Parque Nacional da Serra dos Órgãos ( *Phasmahyla guttata* ) and one species globally disappeared since 1968 (*Phantasmarana bocainensis*) (Figure 1 and Table 1). Additional details are provided in Lopes et al. (2021, 2023).

The linear distance between the southernmost (Lauro Müller) and northernmost (Parque Nacional da Serra do Cipó) sampling locations was 1 183.04 km. The number of samples collected per site (3–16), the perimeter (0.37–68.01 km) and the polygon area (0.27–193.04 km^2^) (Appendix S1) of the surveyed sites were proportional to the number of known exact spots of historical occurrence and the availability of suitable habitats for our targeted species.

A total of 12,141,610 raw sequence reads were obtained from the 117 eDNA samples: 11 negative and six positive controls for our *Bd* dataset, which included the combined NBAF and SBAF samples. Lineages were assigned to all 33 *Bd* sequences recovered from the combined eDNA samples: 15 were identified as the local enzootic *Bd*‐Brazil, and the remaining 18 sequences were identified as the global panzootic *Bd* lineage (*Bd*‐GPL). DNA traces of *Bd* were detected at all NBAF and SBAF localities surveyed, with the exception of Nova Friburgo and the adjacent Cerrado grasslands at Parque Nacional da Serra do Cipó (Figure 1 and Appendix S1). DNA traces of *Bd*‐GPL and *Bd*‐Brazil were detected in canopy‐covered microhabitats, such as streams, rivers, rocky seeps, and bromeliads. In contrast, no DNA traces of *Bd* were found in open‐canopy still water bodies, such as puddles, ponds, or swamps (Appendix S1). *Bd*‐GPL was the only lineage detected in the northernmost (Santa Teresa) and southernmost localities (Águas Mornas, and Lauro Müller) and at Parque Nacional da Serra da Bocaina (Figure 1 and Appendix S1 and S4). In our more centrally located sites (Parque Nacional da Serra dos Órgãos, Parque Nacional de Itatiaia, Estação Biológica de Boracéia, Morretes, São Bento do Sul, and Rio dos Cedros) we detected DNA traces of both *Bd*‐GPL and *Bd*‐Brazil, with a significantly greater number of reads and positive samples of *Bd*‐GPL at all sites (Wilcoxon's *p* < 0.01, Appendix S5). The relative frequency of *Bd*‐GPL reads was enriched at all sampling sites (from 1.37‐ to 41.10‐fold) compared to *Bd*‐Brazil (Appendix S6). At Morretes, we observed the highest number of positive samples (7) and read counts (190442) for *Bd*‐Brazil (Appendix S4).

The estimated false positive rates (p10) for each target amphibian species and *Bd* lineage detected ranged from 0.003–0.008. The probability of detecting the DNA of a target amphibian in the environment (p11) was 0.139–0.949. For *Bd*‐GPL and *Bd*‐Brazil, p11 = 0.346 and 0.633, respectively. The estimated proportion of sites occupied (psi) for target amphibian species ranged from 0.097–0.624, while for *Bd*‐GPL and *Bd*‐Brazil, psi = 0.441 and 0.200, respectively (Table 2).

**TABLE 2:** Estimated values of false positive rates (p10), probability of species detection if present in the environment (p11) and occupancy of sites (psi) for *Bd* lineages and target amphibian species positively detected using eDNA in this ( *Boana semiguttata* and *Cycloramphus cedrensis* ) and previously published studies ( *Hylodes ornatus* , *Hylodes regius* , *Crossodactylus timbuhy* , *Vitreorana eurygnatha* , *Phasmahyla exilis* , *Phasmahyla guttata* , and *Phantasmarana bocainensis*).

	p10	p11	psi
*Hylodes ornatus*	0.003 (0.000–0.009)	0.948 (0.750–0.998)	0.097 (0.015–0.287)
*Hylodes regius*	0.003 (0.000–0.009)	0.250 (0.049–0.537)	0.107 (0.015–0.368)
*Phantasmarana bocainensis*	0.005 (0.000–0.010)	0.337 (0.194–0.504)	0.585 (0.220–0.895)
*Crossodactylus timbuhy*	0.005 (0.000–0.010)	0.723 (0.631–0.810)	0.624 (0.375–0.834)
*Phasmahyla exilis*	0.005 (0.000–0.010)	0.300 (0.081–0.595)	0.132 (0.019–0.413)
*Vitreorana eurygnatha*	0.004 (0.000–0.010)	0.139 (0.027–0.360)	0.224 (0.044–0.743)
*Phasmahyla guttata*	0.005 (0.000–0.010)	0.189 (0.004–0.957)	0.103 (0.003–0.852)
*Boana semiguttata*	0.004 (0.000–0.010)	0.398 (0.266–0.537)	0.412 (0.168–0.691)
*Cycloramphus cedrensis*	0.008 (0.004–0.010)	0.949 (0.743–0.998)	0.314 (0.053–0.721)
*Bd*_GPL	0.004 (0.000–0.017)	0.633 (0.559–0.704)	0.441 (0.325–0.534)
*Bd*_Brazil	0.008 (0.000–0.019)	0.346 (0.210–0.514)	0.200 (0.105–0.334)

*Note:* 95% confidence intervals are shown in parentheses.

We detected a positive correlation between target amphibian DNA and *Bd*‐GPL in filtered water samples (*r* = 0.315, *p* = 0.035), as well as links between several environmental variables (Appendices S7 and S8). Generalised Linear Mixed Models indicate that annual precipitation and elevation were key predictors of DNA traces of our target amphibian species, with target amphibians being more likely to occur at high elevation sites and in areas with relatively low precipitation (Table 3, Appendix S9). The presence of *Bd*‐GPL was also a positive predictor of DNA traces of our target amphibians in the same model, although this result could be an artefact of matching environmental conditions for both hosts and *Bd*. Indeed, when the detection of *Bd*‐GPL or *Bd*‐Brazil was included in separate models, treating it as a single response variable rather than as a predictor variable, the presence of target amphibian DNA in water samples did not demonstrate statistical significance as a predictor. In both models, *Bd*‐GPL and *Bd*‐Brazil showed higher occurrence likelihood at high elevation sites and in areas with relatively low precipitation (Table 3). *Bd*‐GPL detection was more prevalent in filtered samples characterised by smaller water volumes. All final models showed low cross‐correlation among explanatory variables (VIF < 4).

**TABLE 3:** Best‐fit Generalised Linear Mixed Model (GLMMs) with Logistic Link predicting presence of target amphibian DNA or *Bd* lineage. Sampling site and target amphibian species searched were both included as random factors in all models. Number of samples (capsules) per sampling site was also accounted for in the analyses.

Explanatory variable	Estimate	Std. Error	*z* value	VIF	*p*
*Response variable: presence of target amphibian DNA in water samples*					
*Bd‐*GPL	8.183	4.039	2.026	1.578	0.042*
Elevation	0.004	0.002	2.139	3.048	0.034*
Annual Rainfall	−0.008	0.004	−2.282	2.459	0.022*
*Response variable: presence of Bd‐GPL in water samples*					
Elevation	−0.001	0.001	−2.287	1.01	0.022*
Filtered Volume	−0.002	0.001	−2.245	1.01	0.014*
*Response variable: presence of Bd‐Brazil in water samples*					
Elevation	−0.004	0.002	−2.154	1.931	0.031*
Annual Rainfall	0.01	0.005	2.086	1.931	0.037*

*Note:* Best‐fit “pruned” models for each response variable were based on Akaike Information Criterion (AIC). Std. Error stands for standard error.

* Statistically significant (*p* < 0.05).

## 4 Discussion

Our results highlight the effectiveness of water‐based eDNA sampling in simultaneously monitoring the presence of threatened aquatic‐breeding amphibian species and the fungal pathogen *Bd* in the environment. We successfully detected two of our 10 targeted amphibian species from the SBAF: *Boana semiguttata* at the type locality of Rio Vermelho (Santa Catarina) (Table 1), and *Cycloramphus cedrensis* at the type locality of Rio dos Cedros municipality (Santa Catarina) (Table 1). From the aggregated survey data (SBAF and NBAF), we detected DNA traces of two *Bd* lineages (*Bd*‐GPL and *Bd*‐Brazil) out of the five major lineages described to date (O'Hanlon et al. 2018). The distributions of the two *Bd* lineages varied by location, with *Bd* lineages being undetected in samples obtained from open‐canopy still‐water bodies, such as puddles, ponds, or swamps.

Natural population fluctuations, low population abundances, and incomplete detection could explain why some of our target species were not detected in traditional surveys over the last few decades (Eterovick et al. 2005). The high accuracy of eDNA metabarcoding can partially overcome the challenge of false negatives, allowing us to survey species at all life stages (e.g., eggs, tadpoles, or adults [in the case of amphibians], zoospores and hyphae [in the case of *Bd*]), and multiple taxonomic groups present within the same sample (Taberlet et al. 2018). These advantages reinforce the importance of integrating eDNA metabarcoding into biodiversity monitoring programmes (Osman et al. 2022). However, as with any survey method, eDNA metabarcoding is also prone to imperfect detections of target species. The batra primers demonstrated high specificity for amplifying the DNA of our target amphibians and provided good taxonomic resolution at the species level. However, in cases of poor taxonomic resolution and species boundaries, as we show with the case of *Cycloramphus cedrensis* , it is possible to not arrive at a full species diagnosis based exclusively on the metabarcoding fragment. Nonetheless, to ensure high standards of quality control and prevent uncertainties arising from sampling design and associated laboratory assays (Taberlet et al. 2018), estimated probabilities from site occupancy models may be used to infer the reliability of presence/absence data for targeted species (Willoughby et al. 2016). The low levels of false‐positive rates estimated from our data support the high accuracy of our target DNA detection from both amphibians and *Bd* lineages.

Our findings highlight that environmental *Bd* is relatively widespread across the Brazilian Atlantic forest (Figure 1), with the Global Panzootic *Bd* lineage (*Bd*‐GPL) occurring in almost all our focal sampling sites. Interestingly, *Bd*‐Brazil had a more restricted distribution, mainly concentrated in the central region of the Atlantic forest. This agrees with previous findings using host detection methods (Jenkinson et al. 2016). Amphibian species and *Bd* have co‐occurred in the Brazilian Atlantic forest for over a century, with historical records of *Bd*‐GPL and *Bd*‐Brazil dating back to 1894 and 1916, respectively (Rodriguez et al. 2014). Therefore, it is possible that the presence of *Bd* has already impacted populations in the past (Voyles et al. 2018). Hence, species that are highly susceptible to chytridiomycosis may have already disappeared, while mechanisms of *Bd* resistance or tolerance more likely evolved in species with lower initial *Bd* susceptibility (Wilber et al. 2017). Our 42 target threatened amphibians are distributed in eight families, especially in Cycloramphidae, Hylidae, and Hylodidae, with a predominance of montane stream‐breeding species. However, the detection of *Bd* is widespread in other host families, including pond and terrestrial breeders (Rodriguez et al. 2014). This emphasises the need to establish protocols which are applicable for leaf litter or soil samples, in order to understand the impact of pathogens such as *Bd* on terrestrial‐breeding amphibians (Lopes et al. 2021).

Our 42 target species were mainly distributed in protected areas of pristine and continuous forest which were established before or during the amphibian crisis of 1976–1985 (Appendix S1). Hence, it is unlikely that environmental degradation, such as habitat fragmentation, pollution, or even overexploitation could explain the observed major declines and disappearance of amphibians. The introduction of *Bd*‐GPL to the Atlantic forest, and perhaps the coinfection and/or hybridisation with *Bd*‐Brazil (Jenkinson et al. 2016; Greenspan et al. 2018), are acknowledged as a potential driver of these declines, evident from the nature and timing of population disappearances (Carvalho et al. 2017; Scheele et al. 2019). Our findings add credibility to the role of *Bd* being a major cause for the loss of amphibian diversity in Brazil, with both *Bd*‐GPL and *Bd*‐Brazil being detected in the Atlantic Forest sites with the highest records of amphibian population declines and presumed extinctions, such as Estação Biológica de Boracéia, Parque Nacional do Itatiaia, Parque Nacional da Serra dos Órgãos, and Santa Teresa. Given that *Bd* is a host‐generalist pathogen among amphibians, and that pristine, highly connected habitats support higher amphibian diversity and optimal microclimatic conditions for *Bd* survival and spread (Becker and Zamudio 2011), it is not surprising that we only found *Bd* DNA traces in closed‐canopy microhabitats such as streams, rivers, rocky seeps, and bromeliads; with *Bd* remaining undetected in warmer open‐canopy still water bodies, such as puddles, ponds, or swamps.

Although we found a positive association between *Bd*‐GPL in the environment and the likelihood of detecting the DNA of target amphibians, other environmental variables were also significant predictors in the same GLMM and pairwise correlations. While rainfall and temperature (elevation) were key predictors of target amphibian DNA in best‐fit GLMMs, it is likely that they are not capturing the wide range of microclimatic variation in these habitats, and that *Bd*‐GPL might contribute as a better proxy for optimal amphibian microclimatic conditions. In fact, our pairwise correlations indicated that *Bd*‐GPL was highly correlated with elevation. Other population surveys across elevational gradients confirm this pattern; *Bd* infection‐prevalence in Andean amphibians increased when host temperatures matched *Bd* optimal growth range (Catenazzi et al. 2014). New research also indicates that the presence of alternative non‐amphibian *Bd* reservoirs or vectors, such as waterfowl, squamate reptiles, fishes, and crustaceans, could be an important unmeasured variable explaining observed patterns of pathogen occurrence (Prahl et al. 2020), but almost nothing is known about alternative *Bd* hosts in tropical regions. Our results indicate a post‐pandemic equilibrium scenario between amphibians and *Bd*, with rare species coexisting with *Bd* lineages varying in virulence (Greenspan et al. 2018). Amphibian populations that were once deemed extinct are now being rediscovered in the Neotropics and Australia (Scheele et al. 2019; Toledo et al. 2023). Our rediscoveries in Brazil are a glimmer of hope in a time characterised by rapid global change and increasing biodiversity loss.

## Author Contributions

C.M.L., D.B., J.P.C.M., and C.F.B.H. designed research; C.G.B., A.C., K.R.Z., and C.F.B.H. acquired financial support; C.M.L., D.B., and J.P.C.M. completed field sampling; C.M.L. and M.L.L. did the laboratory work; C.M.L., C.G.B., and M.L.L. analyzed the data; C.M.L. wrote the manuscript with contributions from all coauthors.

## Conflicts of Interest

The authors declare no conflicts of interest.

## Supporting information


**Appendix S1:** mec70075‐sup‐0001‐AppendixS1.pdf.

## Data Availability

NGS eDNA unfiltered data for NBAF (https://doi.org/10.5061/dryad.f4qrfj6tc) and SBAF amphibian and *Bd* datasets, as well as bioinformatic pipelines (https://datadryad.org/stash/share/ueUEipLVBGB45Y9abHERoXLWdjPeYnjEdfUzJhINObE) are deposited in Dryad. DNA sequences of 12S rRNA mitochondrial gene of amphibians for the local reference database are deposited in GenBank (OR450105‐OR450259).
