# Supplementary material for: Simultaneous Detection of Missing Amphibians and Their Fungal Pathogen in a Biodiversity Hotspot Using eDNA
Source: Mol Ecol. 2025 Aug 8;34(19):e70075. doi: 10.1111/mec.70075 (PMC12456115; doi:10.1111/mec.70075)
Supplement: Supplementary file 1 — Appendix S1: mec70075‐sup‐0001‐AppendixS1.pdf. [file MEC-34-e70075-s001.pdf]

Supporting Information for:

Simultaneous detection of missing amphibians and their fungal pathogen in a biodiversity hotspot using eDNA

Table of Contents:

**Appendix S1**	Pages 2-7
**Appendix S2**	Page 8
**Appendix S3**	Pages 9-10
**Appendix S4**	Page 11
**Appendix S5**	Page 12
**Appendix S6**	Page 13
**Appendix S7**	Page 14
**Appendix S8**	Page 15
**Appendix S9**	Page 16

Appendix S1. Summary information for eDNA samples. Sites previously sampled by Lopes *et al*. (2021 and 2023) (northern Brazilian Atlantic forest - NBAF) and sampled for this study (southern Brazilian Atlantic forest – SBAF) with abbreviation of states, perimeter (km) and area (km^2) of the polygon surveyed, name of the protected area if samples were collected inside one, date of creation of the protected area, sampling identification, date of sampling, volume of water filtered, source of water sampled, sampling strategy used in each sampling site, geographical coordinates, elevation in meters, and *Batrachochytrium dendrobatidis* lineages or target amphibian species with conservation concern positively detected using eDNA are shown.

Sampling site	Protected Area	Date of creation	Sample ID	Date	Volume	Source	Strategy	Geographical coordinate	Elevat.	eDNA positive detections
Parque Nacional da Serra do Cipó – MG (NBAF)	Parque Nacional da Serra do Cipó	1984 www.icmbio.gov.br/parnaserradocipo/quem-somos/decreto-de-criacao	WF151109	11/02/2016	2 L	Puddle	VigiDNA	S -19.257250; W -43.532639	1353	NA
			WF151156	11/02/2016	2 L	Puddle	VigiDNA	S -19.257250; W -43.532639	1353	NA
			SPY160008	03/12/2015	20 L	Puddle	Envirochek	S -19.257250; W -43.532639	1370	NA
Santa Teresa – ES (NBAF) 35,85 km 24,68 km^2	-	-	WF151155	23/02/2016	30 L	Stream	VigiDNA	S -19.973611; W -40.529028	582	NA
	-	-	WF151142	23/02/2016	15 L	Stream	VigiDNA	S -19.967250; W -40.536167	614	NA
	-	-	WF151139	26/02/2016	2 L	Puddle	VigiDNA	S -19.965722; W -40.539778	631	NA
	Reserva Biológica Augusto Ruschi	1982 www.diariodasleis.com.br/legislacao/federal/34062-denomina-augusto-ruschi-a-reserva-biologica-de-nova-lombardia-no-municipio-de-santa-tereza-estado-do-espirito-santo	WF151149	24/02/2016	30 L	Stream	VigiDNA	S -19.910833; W -40.554583	825	*Bd*-GPL and *Crossodactylus timbuhy*
			WF151148	24/02/2016	30 L	Rocky seep	VigiDNA	S -19.925194; W -40.555861	784	*Crossodactylus timbuhy*
			WF151146	24/02/2016	25 L	Stream	VigiDNA	S -19.880944; W -40.540528	812	*Bd*-GPL, *Crossodactylus timbuhy*, and *Phasmahyla exilis*
			WF151144	24/02/2016	30 L	Stream	VigiDNA	S -19.882722; W -40.541667	797	*Crossodactylus timbuhy* and *Phasmahyla exilis*
			WF151141	25/02/2016	4 L	Pond	VigiDNA	S -19.883972; W -40.542278	777	*Crossodactylus timbuhy*
			WF151151	25/02/2016	30 L	Stream	VigiDNA	S -19.904972; W -40.550944	799	*Bd*-GPL, *Crossodactylus timbuhy*, and *Vitreorana eurygnatha*
			WF151137	25/02/2016	30 L	Stream	VigiDNA	S -19.910583; W -40.542056	813	*Crossodactylus timbuhy*
			WF151143	25/02/2016	30 L	Stream	VigiDNA	S -19.909306; W -40.542167	812	*Bd*-GPL and *Crossodactylus timbuhy*
			WF151150	25/02/2016	30 L	Stream	VigiDNA	S -19.908028; W -40.547806	811	*Crossodactylus timbuhy*

	-	-	WF151138	25/02/2016	30 L	Stream	VigiDNA	S -19.923806; W -40.621444	801	*Bd*-GPL and Vitreorana eurygnatha
	-	-	WF151145	25/02/2016	30 L	Stream	VigiDNA	S -19.924083; W -40.621417	779	NA
Nova Friburgo – RJ (NBAF) 6,94 km 2,28 km^2	Área de Proteção Ambiental Estadual de Macaé de Cima	2001 www.inea.rj.gov.br/biodiversidade-territorio/conheca-as-unidades-de-conservacao/apa-de-macae-de-cima/	SPY171141	07/02/2017	45 mL	Bromeliads	Precipitation	S -22.43; W -42.53	~1100	NA
			SPY171142	08/02/2017	1500 mL	Bromeliads	Precipitation	S -22.43; W -42.51	~1180	NA
			SPY171143	08/02/2017	1500 mL	Bromeliads	Precipitation	S -22.43; W -42.51	~1180	NA
			SPY171144	08/02/2017	90 mL	Bromeliads	Precipitation	S -22.41; W -42.52	~1100	NA
Parque Nacional da Serra dos Órgãos – RJ (NBAF) 16,27 km 6,13 km^2	Parque Nacional da Serra dos Órgãos	1939 www.icmbio.gov.br/parnaserradosorgaos/quem-somos/historia	WF151130	26/03/2016	30 L	Stream	VigiDNA	S -22.448944; W -43.004278	1165	NA
			WF151140	26/03/2016	30 L	Stream	VigiDNA	S -22.448167; W -43.004972	1227	NA
			WF151135	26/03/2016	30 L	River	VigiDNA	S -22.447331; W -43.005550	1249	*Phasmahyla guttata*
			WF151132	26/03/2016	30 L	Stream	VigiDNA	S -22.448250; W -43.007194	1289	NA
			WF151129	26/03/2016	30 L	Stream	VigiDNA	S -22.455167; W -42.999167	1157	*Bd*-GPL
			WF151131	26/03/2016	30 L	River	VigiDNA	S -22.456539; W -42.997400	1106	NA
			WF151127	26/03/2016	30 L	River	VigiDNA	S -22.448531; W -43.013419	1477	NA
			SPY1600992	26/03/2016	30 L	River	Envirochek	S -22.493472; W -42.999083	402	NA
			WF151134	26/03/2016	30 L	Stream	VigiDNA	S -22.494181; W -43.000711	395	NA
			WF151133	26/03/2016	4 L	Rocky seep	VigiDNA	S -22.474769; W -42.999169	825	NA
			WF151136	27/03/2016	4 L	Rocky seep	VigiDNA	S -22.463861; W -42.990444	948	Na
			WF151128	27/03/2016	4 L	Rocky seep	VigiDNA	S -22.458972; W -42.977944	984	*Bd*-GPL
			SPY171138	05/02/2017	1500 mL	Bromeliads	Precipitation	S -22.45; W -42.99	~1050	*Bd*-Brazil
			SPY171139	05/02/2017	1500 mL	Bromeliads	Precipitation	S -22.45; W -42.99	~1040	NA
			SPY171140	06/02/2017	1500 mL	Bromeliads	Precipitation	S -22.45; W -42.99	~1100	*Bd*-GPL
		1937	WF151125	31/01/2016	30 L	Stream	VigiDNA	S -22.364139; W -44.721917	2245	*Bd*-GPL

Parque Nacional de Itatiaia – RJ (NBAF) 12,53 km 3,37 km^2	Parque Nacional de Itatiaia	www.icmbio.gov.br/parnaitatiaia/qm-somos	WF151110	01/02/2016	30 L	Stream	VigiDNA	S -22.379167; W -44.690667	2392	NA
			WF151123	01/02/2016	30 L	Stream	VigiDNA	S -22.378389; W -44.691667	2409	NA
			WF151116	01/02/2016	30 L	Stream	VigiDNA	S -22.365889; W -44.716389	2339	*Bd*-GPL and *Bd*-Brazil
			WF151102	01/02/2016	30 L	Stream	VigiDNA	S -22.366222; W -44.715000	2346	NA
			WF151104	01/02/2016	30 L	Stream	VigiDNA	S -22.363139; W -44.727750	2262	NA
			WF151103	01/02/2016	30 L	Swamp	VigiDNA	S -22.358972; W -44.737167	2165	NA
			WF151112	01/02/2016	30 L	Stream	VigiDNA	S -22.365694; W -44.745611	1952	*Bd*-GPL and *Hylodes regius*
			WF151098	01/02/2016	30 L	Stream	VigiDNA	S -22.362528; W -44.734917	2059	*Bd*-GPL and *Hylodes ornatus*
			WF151106	02/02/2016	30 L	Stream	VigiDNA	S -22.364139; W -44.721917	2245	NA
			WF151152	02/02/2016	30 L	Stream	VigiDNA	S -22.363083; W -44.727806	2230	NA
			WF151154	02/02/2016	30 L	Stream	VigiDNA	S -22.379750; W -44.691000	2408	NA
			WF151153	02/02/2016	30 L	River	VigiDNA	S -22.377694; W -44.693611	2411	NA
			WF151100	02/02/2016	20 L	Stream	VigiDNA	S -22.377722; W -44.693194	2415	NA
			WF151105	02/02/2016	30 L	Stream	VigiDNA	S -22.364333; W -44.723583	2294	NA
			WF151101	02/02/2016	30 L	Stream	VigiDNA	S -22.370833; W -44.705917	2387	NA
Parque Nacional da Serra da Bocaina – SP (NBAF) 0,37 km 0,89 km^2	Parque Nacional da Serra da Bocaina	1971 www.icmbio.gov.br/parnaserradabocaina/quem-somos/decretos-de-criacao	WF151147	04/02/2016	30 L	River	VigiDNA	S -22.731306; W -44.596222	1511	*Bd*-GPL and *Phantasmarana bocainensis*
			WF151107	04/02/2016	30 L	River	VigiDNA	S -22.731000; W -44.596472	1512	*Bd*-GPL and *Phantasmarana bocainensis*
			WF151111	04/02/2016	30 L	River	VigiDNA	S -22.731389; W -44.596056	1515	*Bd*-GPL and *Phantasmarana bocainensis*
			WF151099	04/02/2016	2 L	Bromeliads	VigiDNA	S -22.731306; W -44.596222	1500	NA
			WF151108	04/02/2016	30 L	Stream	VigiDNA	S -22.729861; W -44.596556	1492	*Bd*-GPL
Estação Biológica de Boracéia – SP (NBAF) 10,96 km	Estação Biológica de Boracéia	1954 mz.usp.br/pt/pesquisa/estacao-	WF151120	17/01/2016	10 L	Rocky seep	VigiDNA	S -23.638111; W -45.840333	877	*Bd*-GPL and *Bd*-Brazil
			WF151121	17/01/2016	30 L	Stream	VigiDNA	S -23.637944; W -45.839889	859	NA

0,89 km^2		biologica-de-boraceia	WF151114	17/01/2016	30 L	Stream	VigiDNA	S -23.640111; W -45.847611	840	*Bd*-GPL
			WF151115	17/01/2016	30 L	Stream	VigiDNA	S -23.646278; W -45.864611	872	*Bd*-GPL and *Bd*-Brazil
			WF151113	17/01/2016	30 L	Stream	VigiDNA	S -23.650750; W -45.871917	869	NA
			WF151118	17/01/2016	30 L	Stream	VigiDNA	S -23.652972; W -45.890028	840	NA
			WF151122	18/01/2016	30 L	Stream	VigiDNA	S -23.637944; W -45.839889	859	NA
			WF151117	18/01/2016	10 L	Rocky seep	VigiDNA	S -23.637861; W -45.840667	878	*Bd*-GPL
			WF151119	18/01/2016	30 L	Stream	VigiDNA	S -23.651028; W -45.871972	870	*Bd*-GPL
			WF151126	18/01/2016	30 L	Stream	VigiDNA	S -23.652056; W -45.876056	884	NA
			WF151097	18/01/2016	30 L	Stream	VigiDNA	S -23.640250; W -45.847417	869	*Bd*-GPL and *Bd*-Brazil
			WF151124	19/01/2016	30 L	Stream	VigiDNA	S -23.654222; W -45.888639	841	*Bd*-GPL and *Bd*-Brazil
Morretes – PR (SBAF) 27,61 km 23,58 km^2	Área de Especial Interesse Turístico do Marumbi	1984 paranainterativo.pr.gov.br/portal/apps/webappviewer/index.html?id=a3c402165af04809a3c8c0f79d5cfc4c	MOR_01	08/04/2019	180 L	Stream	Envirochek	S -25.34220; W -48.92191	897	*Bd*-GPL and *Bd*-Brazil
			MOR_02	08/04/2019	120 L	Stream	Envirochek	S -25.341252; W -48.918561	902	*Bd*-Brazil
			MOR_03	08/04/2019	90 L	Stream	Envirochek	S -25.333388; W -48.90768	911	NA
			MOR_04	08/04/2019	120 L	River	Envirochek	S -25.33745; W -48.90792	928	NA
			MOR_05	08/04/2019	120 L	Stream	Envirochek	S -25.35219; W -48.88698	552	*Bd*-GPL and *Bd*-Brazil
	Parque Estadual Pico do Marumbi	1990 paranainterativo.pr.gov.br/portal/apps/webappviewer/index.html?id=a3c402165af04809a3c8c0f79d5cfc4c	MOR_06	09/04/2019	120 L	River	Envirochek	S -25.44190; W -48.92283	574	*Bd*-GPL and *Bd*-Brazil
			MOR_07	09/04/2019	120 L	River	Envirochek	S -25.44564; W -48.92484	659	NA
			MOR_08	09/04/2019	120 L	Stream	Envirochek	S -25.44497; W -48.92482	663	*Bd*-GPL and *Bd*-Brazil
			MOR_09	09/04/2019	120 L	Stream	Envirochek	S -25.44246; W -48.92368	598	*Bd*-GPL and *Bd*-Brazil
	Área de Especial Interesse Turístico do Marumbi	1984 paranainterativo.pr.gov.br/portal/apps/webappviewer/index.html?id=a3c402165af04809a3c8c0f79d5cfc4c	MOR_10	10/04/2019	120 L	Stream	Envirochek	S -25.33417; W -48.89895	816	*Bd*-GPL and *Bd*-Brazil

São Bento do Sul – SC (SBAF) 68,01 km 193,04 km^2	Área de Proteção Ambiental Serra Dona Francisca	1997	SBS_01	11/04/2019	120 L	Stream	Envirochek	S -26.24980; W -49.26008	942	*Boana semiguttata*
			SBS_02	11/04/2019	40 L	Stream	Envirochek	S -26.24667; W -49.25410	953	*Bd*-GPL, *Bd*-Brazil, and *Boana semiguttata*
	Área de Proteção Ambiental do Rio Vermelho - Humboldt	1998	SBS_03	11/04/2019	120 L	Stream	Envirochek	S -26.25348; W -49.26904	944	*Bd*-GPL and *Bd*-Brazil
			SBS_04	12/04/2019	60 L	Stream	Envirochek	S -26.34824; W -49.28189	205	*Bd*-GPL
			SBS_05	12/04/2019	96 L	Stream	Envirochek	S -26.35278; W -49.28027	203	*Bd*-GPL and *Bd*-Brazil
	Estação Ecológica do Bracinho	1984	SBS_06	19/04/2019	60 L	River	Envirochek	S -26.28650; W -49.11542	613	*Bd*-GPL
	Área de Proteção Ambiental Serra Dona Francisca	1997	SBS_07	19/04/2019	120 L	Stream	Envirochek	S -26.23317; W -49.05423	821	*Bd*-GPL and *Boana semiguttata*
			SBS_08	20/04/2019	120 L	Stream	Envirochek	S -26.23317; W -49.05423	821	*Bd*-GPL, *Bd*-Brazil, and *Boana semiguttata*
			SBS_09	20/04/2019	120 L	Stream	Envirochek	S -26.22537; W -49.05719	735	NA
			SBS_10	20/04/2019	120 L	Stream	Envirochek	S -26.19175; W -49.02977	425	*Bd*-GPL and *Bd*-Brazil
Rio dos Cedros – SC (SBAF) 44,98 km 13,91 km^2	-	-	CED_01	12/04/2019	54 L	Stream	Envirochek	S -26.55943; W -49.42093	880	*Bd*-GPL and *Bd*-Brazil
	-	-	CED_02	12/04/2019	70 L	Stream	Envirochek	S -26.65374; W -49.35855	606	*Bd*-GPL, *Bd*-Brazil, and *Cycloramphus*
	-	-	CED_03	13/04/2019	72 L	Stream	Envirochek	S -26.74224; W -49.32446	533	*Bd*-GPL, *Bd*-Brazil, and *Cycloramphus*
	-	-	CED_04	13/04/2019	70 L	Stream	Envirochek	S -26.73894; W -49.32301	495	*Cycloramphus*
Águas Mornas – SC (SBAF) 32,39 km 56,38 km^2	Parque Estadual da Serra do Tabuleiro	1975	AMO_01	14/04/2019	120 L	Stream	Envirochek	S -27.83066; W -48.96477	764	*Bd*-GPL
	-	-	AMO_02	14/04/2019	94 L	Stream	Envirochek	S -27.85752; W -48.95509	508	NA
	Parque Estadual da Serra do Tabuleiro	1976	AMO_03	14/04/2019	52 L	Stream	Envirochek	S -27.79746; W -48.96512	335	*Bd*-GPL
			AMO_04	15/04/2019	120 L	Stream	Envirochek	S -27.80202; W -48.96428	365	*Bd*-GPL
			AMO_05	15/04/2019	120 L	Stream	Envirochek	S -27.81596; W -48.85167	362	*Bd*-GPL
			AMO_06	15/04/2019	120 L	Stream	Envirochek	S -27.81258; W -48.84657	351	*Bd*-GPL

			AMO_07	15/04/2019	120 L	Stream	Envirochek	S -27.81173; W -48.84502	358	*Bd*-GPL
			AMO_08	16/04/2019	22 L	Stream	Envirochek	S -27.77744; W -48.94403	282	NA
			AMO_09	16/04/2019	120 L	Stream	Envirochek	S -27.78741; W -48.95863	358	NA
Lauro Muller – SC (SBAF) 22,36 km 18,08 km^2	-	-	LAS_01	17/04/2019	1020 mL	Rocky seep	Sterivex	S -28.39921; W -49.54980	1300	NA
	-	-	LAS_02	17/04/2019	1020 mL	Rocky seep	Sterivex	S -28.39829; W -49.55054	1080	NA
	-	-	LAS_03	17/04/2019	1080 mL	Rocky seep	Sterivex	S -28.39831; W -49.55014	1286	*Bd*-GPL
	-	-	LAS_04	17/04/2019	1200 mL	Rocky seep	Sterivex	S -28.39926; W -49.54837	1258	*Bd*-GPL
	-	-	LAS_05	17/04/2019	1200 mL	Rocky seep	Sterivex	S -28.39917; W -49.54828	1257	NA
	-	-	LAS_06	17/04/2019	1800 mL	Rocky seep	Sterivex	S -28.39740; W -49.54558	1139	NA
	-	-	LAS_07	17/04/2019	1140 mL	Rocky seep	Sterivex	S -28.39475; W -49.52879	769	NA
	-	-	LAS_08	17/04/2019	1020 mL	Rocky seep	Sterivex	S -28.39428; W -49.52817	767	NA
	-	-	LAU_01	17/04/2019	120 L	Stream	Envirochek	S -28.39528; W -49.52956	767	NA
	-	-	LAU_02	17/04/2019	120 L	Stream	Envirochek	S -28.39685; W -49.54715	1087	NA
	-	-	LAU_03	17/04/2019	120 L	Stream	Envirochek	S -28.39598; W -49.54709	1098	NA
	-	-	LAU_04	17/04/2019	120 L	Stream	Envirochek	S -28.39923; W -49.55000	1302	*Bd*-GPL
	-	-	LAU_05	18/04/2019	120 L	Stream	Envirochek	S -28.37113; W -49.51162	678	*Bd*-GPL
	-	-	LAU_06	18/04/2019	120 L	Stream	Envirochek	S -28.336452; W -49.52102	456	NA
	-	-	LAU_07	18/04/2019	120 L	Stream	Envirochek	S -28.32761; W -49.48283	388	NA

Brazilian states: ES - Espírito Santo; MG - Minas Gerais; PR - Paraná; RJ - Rio de Janeiro; SC - Santa Catarina; SP - São Paulo; Envirochek HV 1 µm - Pall Corporation, Port Washington, USA; VigiDNA 0.45 µm - SPYGEN, Le Bourget-du-Lac, France; Sterivex 0.22 µm - Millipore, Burlington, USA; or precipitation with ethanol and sodium acetate (Lopes et al., 2023). NA – Not assigned.

Appendix S2. Number of Molecular Operational Taxonomic Units (MOTUs) and total read counts retained at each step of the sequence filtering pipeline for amphibian's dataset from southern Brazilian Atlantic forest. Details of the sequence filtering process are described in the supplemental text. The number of eDNA samples analyzed for each site are in parentheses.

	Cons. seq. to PCR replicates	Length > 20pb, Seq. read count ≥ 10	Tax. assign.	Seq. read count ≥ 10	Freq ≥ 0.01	No internals	BI ≥ 96%	Only Anura	No cross contam.	Samples read count ≥ 400	N. taxa
**Total**	1229148/ 11185085	31045 / 9573368	-	-	-	-	-	-	-	-	
**Morretes (N = 10)**	-	-	24458/ 2407988	7115 / 2347793	319 / 1758562	264 / 1713692	61 / 628085	42 / 607551	32 / 602422	19 / 358740	13
**São Bento do Sul (N = 10)**	-	-	25034 / 2763700	8158 / 2702022	323 / 2024217	270 / 1981895	73 / 664426	43 / 470184	35 / 467506	20 / 218819	13
**Rio dos Cedros (N = 4)**	-	-	22755 / 1750561	5839 / 1697030	279 / 1272864	241 / 1246062	65 / 437912	38 / 350127	18 / 255530	8 / 99145	7
**Águas Mornas (N = 9)**	-	-	26323 / 2804345	8812 / 2739347	331 / 2023391	264 / 1979780	58 / 474387	36 / 432038	21 / 423969	8 / 181761	7
**Lauro Müller (N = 15)**	-	-	27977 / 4200898	15223 / 4135035	443 / 2767192	381 / 2678066	56 / 395330	40 / 375284	27 / 361660	13 / 125001	10

Consensus sequences assigned to PCR replicates (Cons. seq. to PCR replicates); sequences longer than 20 bp and with total read counts among all PCR replicates ≥ 10 (Length > 20pb, Seq. read count ≥ 10); sequences taxonomically assigned and separated for each sampling site (Tax. assign.); sequences with total read counts among PCR replicates for each site ≥ 10 (Seq. read count ≥ 10); sequences with a frequency per PCR replicate ≥ 0.001 (Freq ≥ 0.001); sequences identified as 'internal' excluded (No internals); sequences with best identity ≥ 96% with a sequence from the reference database (BI ≥ 96%); sequences classified as Anura (Only Anura); no sequences from cross-contamination sources (No cross contam.); samples ≥ 400 sequence reads count; and number of taxa (tribe, genus, or species) found for each site are shown (N. taxa).

Appendix S3. Amphibian taxa identified in the eDNA samples from southern Brazilian Atlantic forest. The eDNA sequence identification (Seq ID), best identity and best match of eDNA sequences with a sequence from the reference database (represented by GenBank accession numbers), and the number of positive eDNA samples and PCR replicates for each sampling site are shown for each eDNA sequence.

Family	Taxa identification	Seq ID	Best Identity	Best Match	Morretes	São Bento do Sul	Rio do Cedros	Águas Mornas	Lauro Müller
Alsodidae	*Proceratophrys boiei*	seq_00078	1,00	OR450226	2, 19	1, 3	-	-	-
Bufonidae	*Rhinella*	seq_00093	1,00	OR450233	3, 5	-	-	-	3, 9
		seq_00255	1,00	OR450237	1, 7	-	-	-	-
Centrolenidae	*Vitreorana uranoscopa*	seq_04006	1,00	OR450256	-	1, 1	-	-	-
Cycloramphidae	*Cycloramphus*	seq_00016	1,00	OR450150	1, 1	-	3, 15	-	2, 2
		seq_00137	0,98	OR450150	-	1, 11	-	-	-
		seq_02559	0,96	OR450150	1, 1	-	-	-	-
	Cycloramphus rhyakonastes	seq_00192	1,00	OR450158	2, 10	-	-	-	-
Hylidae	*Aplastodiscus*	seq_00094	0,96	MT771821	6, 17	-	-	-	-
	Boana	seq_00036	1,00	OR450117	-	4, 12	2, 6	2, 12	1, 6
	Boana faber	seq_00041	1,00	OR450120	1, 1	-	-	-	1, 11
	Boana semiguttata	seq_00084	1,00	OR450125	-	4, 19	-	-	-
	Cophomantini*	seq_00007	1,00	OR450130	8, 48	3, 4	-	3, 4	-
		seq_00066	0,98	OR450122	-	-	-	2, 14	-
		seq_00311	0,96	OR450130	1, 2	1, 1	-	-	-
		seq_00384	1,00	OR450122	-	1, 1	-	-	-
		seq_00726	0,98	OR450117	-	1, 1	-	-	-
	Dendropsophus minutus	seq_00517	1,00	OR450171	-	-	-	-	2, 2
	Phyllomedusa distincta	seq_01146	1,00	OR450212	-	-	1, 1	-	-
	Ololygon	seq_00188	0,98	MW010332	-	-	-	-	1, 3
		seq_00549	0,98	OQ934178	-	1, 2	-	-	-
	Ololygon catharinae	seq_00032	1,00	OR450203	2, 3	-	-	2, 2	1, 9
		seq_00056	0,98	OR450203	-	2, 13	1, 7	-	-
Hylodidae	Hylodes	seq_00054	1,00	OR450189	-	-	2, 13	-	-
		seq_00015	0,96	OR450189	-	5, 45	2, 13	-	-
		seq_00061	0,98	OR450189	-	1, 5	1, 11	-	-
		seq_00202	0,98	OR450187	-	-	-	1, 1	-
	Hylodes cardosoi	seq_00043	1,00	OR450180	4, 24	-	-	-	-
	Hylodes heyeri	seq_00005	1,00	OR450182	8, 74	5, 36	-	-	-
		seq_00049	0,98	OR450182	2, 15	-	-	-	-
		seq_00050	0,96	OR450182	4, 25	-	-	-	-

		seq_00077	0,98	OR450182	-	2, 20	-	-	1, 1
		seq_00142	0,98	OR450182	1, 5	-	-	-	-
	Hylodes meridionalis	seq_00018	0,98	OR450184	1, 1	-	-	1, 12	-
		seq_00038	0,96	OR450184	-	-	-	-	3, 28
		seq_00298	0,96	OR450184	-	-	-	-	1, 2
		seq_00408	1,00	OR4501843	-	-	-	-	1, 1
	Hylodes perplicatus	seq_00006	1,00	OR450187	-	2, 13	-	6, 48	-
Leptodactylidae	*Adenomera nana*	seq_00236	1,00	OR450110	-	1, 6	-	-	-
		seq_01058	0,98	OR450109	-	-	1, 1	-	-
		seq_02396	0,98	OR450110	-	1, 1	-	-	-
	Leptodactylus	seq_00135	1,00	MT771881	-	1, 11	-	-	-
		seq_00650	0,96	OR450190	-	-	-	-	1, 1
		seq_02524	0,98	MT771881	-	1, 1	-	-	-
	Physalaemus	seq_00646	0,98	OR450219	1, 2	-	-	-	-
	Physalaemus cuvieri	seq_00661	1,00	OR450213	-	-	-	1, 1	-
	Physalaemus lateristriga	seq_00466	1,00	OR450219	1, 3	-	-	-	-

*Environmental DNA sequences assigned to tribe Cophomantini have no genus or species listed. The tribe Cophomantini currently includes the following genera: *Aplastodiscus*, *Boana*, *Bokermannohyla*, *Hyloscirtus*, *Myersiohyla*, and *Nesorohyla*.

Appendix S4. *Batrachochytrium dendrobatidis* (*Bd*) lineages identified in the eDNA samples. The information of localities surveyed (with abbreviation of states), number of eDNA samples analyzed, number of eDNA samples with positive detection for *Bd*-GPL, *Bd*-Brazil and both lineages, and the number of sequence read counts recovered for each lineage in each sampling site are shown.

**Localities**	**N. samples**	**Positive samples**			**Read counts**	
		***Bd*-GPL**	***Bd*-Brazil**	**Total**	***Bd*-GPL**	***Bd*-Brazil**
Parque Nacional da Serra do Cipó – MG	3	-	-	-	-	-
Santa Teresa – ES	14	5	-	5	694222	-
Nova Friburgo – RJ	4	-	-	-	-	-
Parque Nacional da Serra dos Órgãos – RJ	15	3	1	4	252387	41453
Parque Nacional de Itatiaia – RJ	16	4	1	4	231458	5632
Parque Nacional da Serra da Bocaina – SP	5	4	-	4	451807	-
Estação Biológica de Boracéia – SP	12	7	4	7	665607	83895
Morretes – PR	10	6	7	7	260453	190442
São Bento do Sul – SC	10	8	5	8	271201	77646
Rio dos Cedros – SC	4	3	3	3	220077	21508
Águas Mornas – SC	9	6	-	6	459730	-
Lauro Müller – SC	15	4	-	4	410918	-

Brazilian states: ES - Espírito Santo; MG - Minas Gerais; PR - Paraná; RJ - Rio de Janeiro; SC - Santa Catarina; SP - São Paulo.

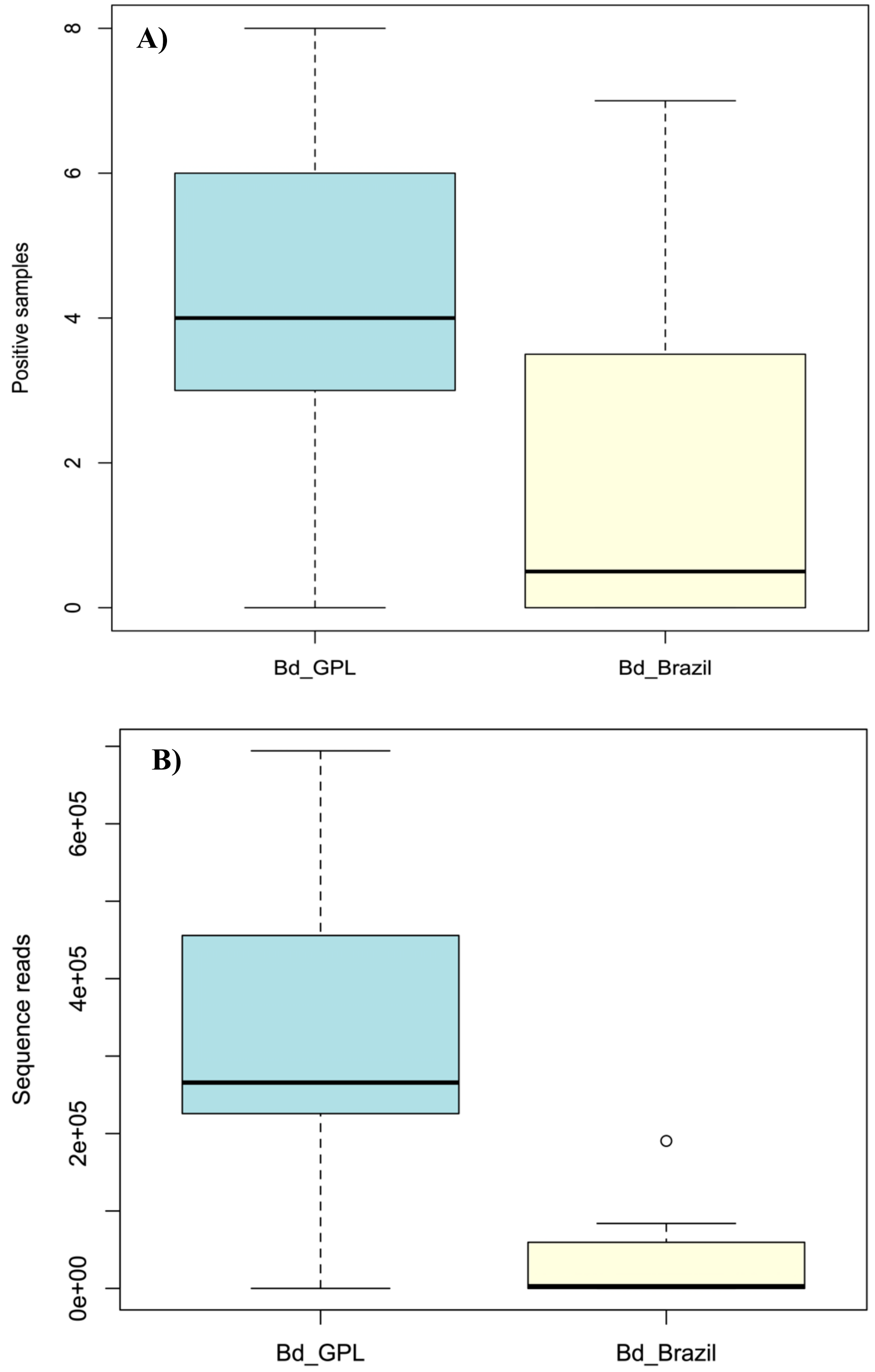

Appendix S5. Boxplot of A) number of sequence reads and B) samples positively detected for the presence of *Bd*-GPL and *Bd*-Brazil lineages considering all localities sampled.

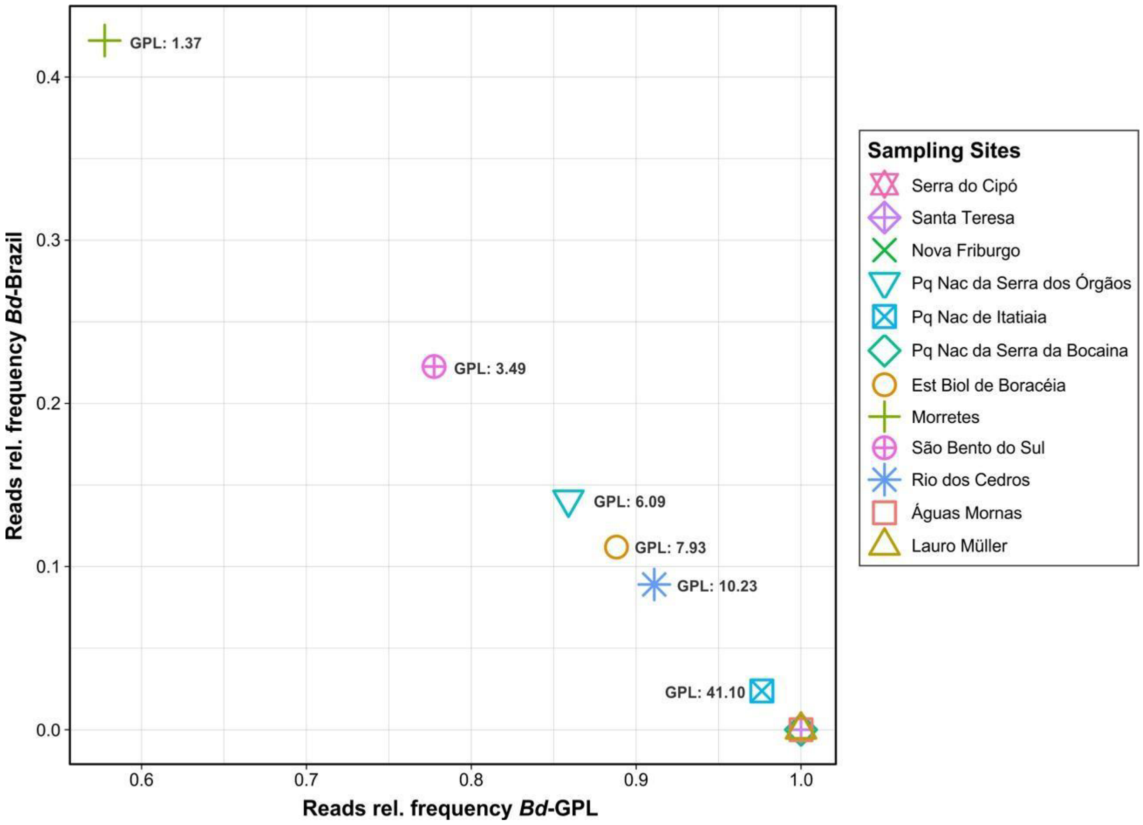

Appendix S6. Relative frequency of sequence read counts compared between *Bd*-GPL and *Bd*-Brazil for each site surveyed in the Brazilian Atlantic forest and adjacent Cerrado grassland. The fold change ratio of the enriched lineage is shown in the plot.

Appendix S7. Pairwise Pearson correlation coefficients (above) and correlation probabilities (below) among focal biotic and abiotic variables. Statistically significant correlations are highlighted in bold.

Correlation coefficients	Target Amphibian DNA	*Bd*-GPL	*Bd*-Brazil	Elevation	Annual Temp.	Annual Rainfall	Filtered Water Volume
Target Amphibian DNA	-						
Bd-GPL	**0.315***	-					
Bd-Brazil	0.175	**0.620**	-				
Elevation	-0.106	**-0.439**	**-0.328**	-			
Annual Temp.	0.081	0.237	0.184	**-0.933**	-		
Annual Rainfall	-0.212	-0.005	0.206	**0.694**	**-0.784**	-	
Filtered Water Volume	-0.127	**-0.412**	-0.17	-0.102	0.049	-0.244	-

Correlation probabilities	Target Amphibian DNA	*Bd*-GPL	*Bd*-Brazil	Elevation	Annual Temp.	Annual Rainfall	Filtered Water Volume
Target Amphibian DNA	-						
Bd-GPL	**0.035**	-					
Bd-Brazil	0.250	**<.0001**	-				
Elevation	0.486	**0.002**	**0.027**	-			
Annual Temp.	0.597	0.116	0.226	**<.0001**	-		
Annual Rainfall	0.161	0.971	0.17	**<.0001**	**<.0001**	-	
Filtered Water Volume	0.406	**0.005**	0.261	0.503	0.748	0.105	-

Appendix S8. Pairwise Pearson correlations among focal biotic and abiotic variables. Lines represent correlation trends without any model adjustment.

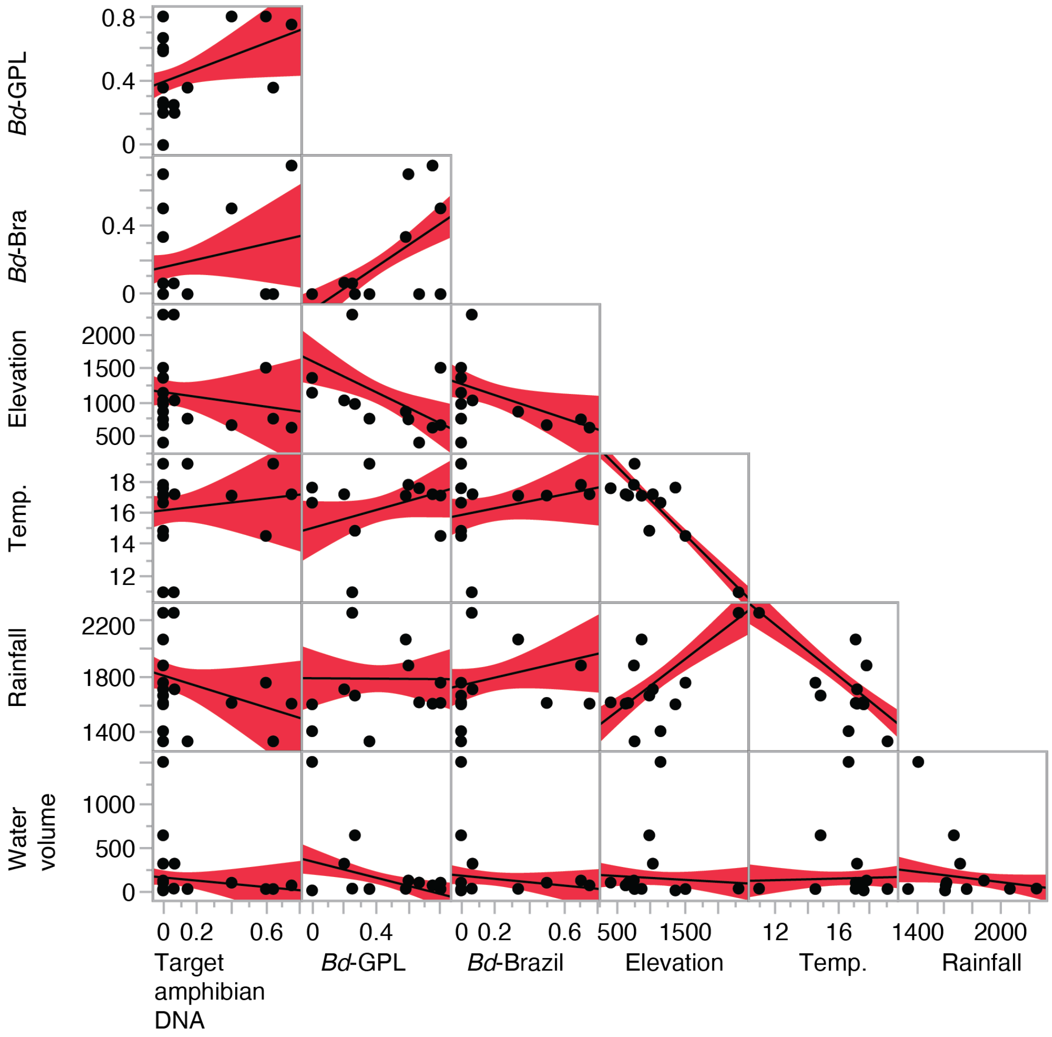

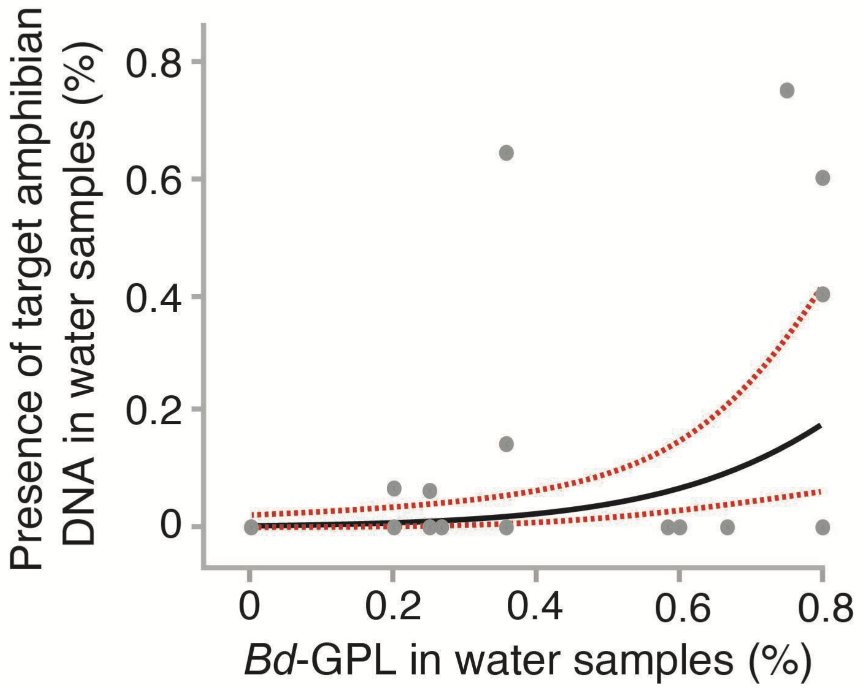

Appendix S9. Relationship between *Bd*-GPL and target amphibian DNA detected in water samples, estimated through the most parsimonious GLMM including two other explanatory variables: elevation and annual rainfall (see Table 3 for full model description).
